# In silico evaluation of Toxoplasma gondii rhoptry neck proteins (TgRONs) for potential immunogenic epitopes

**DOI:** 10.17179/excli2025-8304

**Published:** 2025-07-10

**Authors:** Masoud Forouta, Hany M. Elsheikha, Amir Karimipour-Saryazdi, Ali Dalir Ghaffari, Fatemeh Ghaffarifar, Hamidreza Majidiani

**Affiliations:** 1 Department of Basic Medical Sciences, Faculty of Medicine, Abadan University of Medical Sciences, Abadan, Iran; 2 School of Veterinary Medicine and Science, Faculty of Medicine and Health Sciences, University of Nottingham, Loughborough, LE12 5RD, UK; 3 Department of Parasitology, Faculty of Medical Sciences, Tarbiat Modares University, Tehran, Iran; 4 Department of Parasitology and Mycology, Faculty of Medicine, Shahed University, Tehran, Iran; 5 Healthy Aging Research Centre, Neyshabur University of Medical Sciences, Neyshabur, Iran; 6 Department of Basic Medical Sciences, Neyshabur University of Medical Sciences, Neyshabur, Iran

**Keywords:** in silico analysis, immunoinformatics, rhoptry neck protein, Toxoplasma gondii, vaccine

## Abstract

This immunoinformatics-based study utilized a suite of online predictive tools to characterize the structural and immunogenic properties of *Toxoplasma gondii* rhoptry neck proteins (TgRONs). Full-length amino acid sequences of TgRON2, TgRON4, TgRON4L1, TgRON5, TgRON8, TgRON9, TgRON10, and TgRON13 were retrieved from ToxoDB and subjected to comprehensive analysis. Except for TgRON4L1, all proteins were predicted to be possess antigenic potential, with none identified as allergenic. Solubility predictions indicated that TgRON9 and TgRON10 are the most likely to be expressed as soluble antigens. Aliphatic index values, ranging from 51.17 to 84.63, suggest acceptable thermostability, while negative GRAVY scores across all proteins indicate favorable hydrophilicity. Additionally, multiple post-translational modification sites were identified, underscoring the functional complexity of these antigens. Initial 3D structure modeling showed that 60.21-92.41 % of residues fell within favored regions on Ramachandran plots, with refinement increasing this to 92.27-98.58 %, reflecting substantial improvements in structural quality. Several potential T-cell (CTL and HTL) and B-cell epitopes were predicted for all candidate proteins. Immune simulation models further suggested that these antigens could elicit robust humoral and cellular immune responses when delivered in a three-dose regimen at four-week intervals. These findings offer valuable preliminary insights and support the further investigation of TgRONs, particularly TgRON9 and TgRON10, as promising targets for experimental validation in the development of vaccines against *T. gondii* infection.

See also the graphical abstract(Fig. 1).

## Abbreviations

**3D: **three-dimensional

**AMA1: **apical membrane antigen 1

**ANN: **artificial neural network

**CD: **cluster of differentiation

**CDPK: **calcium-dependent protein kinase

**CTL: **cytotoxic T lymphocyte

**GRA: **dense granule antigen

**GRAVY: **grand average of hydropathicity

**HLA: **human leukocyte antigen

**HTL: **helper T-lymphocyte

**IEDB: **immune epitope database

**IFN-γ: **interferon-γ

**IgG: **immunoglobulin G

**IgM: **immunoglobulin M

**IL-2: **interleukin-2

**kDa: **Kilo Dalton

**MHC: **major histocompatibility complex

**MIC: **microneme protein

**MJ: **moving junction

**MW: **molecular weight

**pI: **isoelectric point

**PTM: **post-translational modification

**RON: **rhoptry neck protein

**ROP: **rhoptry protein

**SAG: **surface antigen

**SVM: **support vector machine

***T. gondii:*** *Toxoplasma gondii*

**TgRONs: ***Toxoplasma gondii* rhoptry neck proteins

**TH: **T-helper cell

## Introduction

*Toxoplasma gondii* (*T. gondii*) is an obligate intracellular protozoan with a global distribution, which causes the zoonotic disease toxoplasmosis. This parasite infects a wide range of intermediate hosts, including humans and warm-blooded vertebrate animals (Dubey, 2008[21]). *T. gondii* infection affects more than one-third of the global population and presents a broad clinical spectrum, ranging from asymptomatic cases to severe or fatal disease (Dubey, 2008[21]; Weiss and Dubey, 2009[87]). In immunocompetent individuals, *T. gondii* infection remains latent and asymptomatic. However, in immunocompromised individuals-such as organ transplant recipients, cancer patients, and those living with HIV-*T. gondii* can cause severe complications, poor clinical outcomes, and even death (Wang et al., 2017[85]; Weiss and Dubey, 2009[87]). Primary infection with *T. gondii* during pregnancy can lead to miscarriage, stillbirth, or a range of birth defects, including intellectual disabilities, blindness, and hydrocephalus (Elsheikha, 2008[23]). 

Current pharmacological treatments primarily target the tachyzoite stage of the parasite during acute infection, but they are ineffective against bradyzoites residing in tissue cysts during the chronic phase (Antczak et al., 2016[2]). Hence, the development of an effective vaccine remains a critical goal for preventing infection in both humans and livestock, given the high global disease burden (Innes et al., 2019[43]). Vaccination offers a promising strategy by inducing long-term protective immunity (McLean, 1998[59]; Yaqub et al., 2014[92]).

Recent research has identified several promising *T. gondii* antigens as potential vaccine candidates. These include microneme proteins (MICs), surface antigens (SAGs), rhoptry proteins (ROPs), dense granule proteins (GRAs), and calcium-dependent protein kinases (CDPKs), which are expressed at various stages of the parasite's life cycle (Foroutan and Ghaffarifar, 2018[27]; Foroutan et al., 2018[31]; Pagheh et al., 2020[62]; Rezaei et al., 2019[66]; Zhang et al., 2013[94]). Immunization studies using these antigens in mouse models have shown partial protection and reduced brain cyst formation following challenge with both virulent and avirulent strains. However, to date, no licensed vaccine is available for human use (Loh et al., 2019[56]; Wang et al., 2019[83]; Zhang et al., 2023[96]).

A key step in the pathogenesis of *T. gondii* is its ability to invade host cells, which begins with the formation of a moving junction (MJ)-a specialized structure formed through intimate contact between the parasite apex and the host cell membrane. This junction is essential for host-cell invasion and parasite motility (Besteiro et al., 2009[6]; Lamarque et al., 2014[50]). The MJ is composed of apical membrane antigen 1 (AMA1), secreted from micronemes, and several rhoptry neck proteins (RONs), secreted from rhoptries (Lamarque et al., 2014[50]). Specifically, RON2, RON4, RON5, and RON8 have been identified as key components of the MJ (Alexander et al., 2005[1]; Straub et al., 2009[78]; Takemae et al., 2013[79]). These proteins play a critical role in host invasion, making them attractive targets for vaccine development. Previous studies have shown that TgRON2, TgRON4, and TgRON5 can elicit strong humoral and cell-mediated immune responses, including increased levels of IFN-γ, IL-2, and IL-4, enhanced survival rates, and reduced tissue cyst burdens in mice (Zhang et al., 2015[95]; Zhao et al., 2016[97]).

In recent years, bioinformatics tools have emerged as powerful approaches for identifying potential vaccine targets by predicting T and B cell epitopes with high antigenic potential (Foroutan et al., 2025[26]; Kazi et al., 2018[46]; Wang et al., 2016[84]). As an interdisciplinary field, bioinformatics integrates methods from biology, physics, statistics, and computer science to analyze and interpret biological data. These tools offer high precision, cost-effectiveness, and efficiency in epitope prediction and structural characterization of vaccine candidates (Flower et al., 2010[25]; Karimipour-Saryazdi et al., 2025[44]; Romano et al., 2011[67]). Accordingly, bioinformatics plays a critical role in the initial stages of vaccine design, enabling the *in silico* evaluation of potential antigens before advancing to experimental validation.

In this study, we employed various online bioinformatics tools to predict structural and immunological features of TgRON proteins, aiming to identify promising epitope candidates for toxoplasmosis vaccine development.

## Materials and Methods

### Source of TgRON protein sequences

The complete amino acid sequences of *T. gondii* rhoptry neck proteins-TgRON2 (TGME49_300100), TgRON4 (TGME49_229010), TgRON4L1 (TGME49_253370), TgRON5 (TGME49_311470), TgRON8 (TGME49_306060), TgRON9 (TGME49_308810), TgRON10 (TGME49_261750), and TgRON13 (TGME49_321650)-were retrieved in FASTA format from the ToxoDB database for bioinformatics analyses (Harb et al., 2020[40]).

### Prediction of physicochemical properties

The SIB Swiss Institute of Bioinformatics is a globally recognized, non-profit organization dedicated to advancing biomedical and biological data science. Its integrative web platform provides access to over 100 tools and databases developed by SIB research groups, supporting diverse fields such as structural biology, proteomics, genomics, systems biology, evolutionary studies, and medicinal chemistry (SIB, Swiss Institute of Bioinformatics Members, 2016[75]). For this study, the ExPASy ProtParam tool was employed to evaluate the physicochemical properties of the TgRON proteins. Parameters analyzed included amino acid composition, molecular weight (MW), chemical formula, theoretical isoelectric point (pI), instability index, aliphatic index, extinction coefficient, grand average of hydropathicity (GRAVY), total number of positively and negatively charged residues, and estimated half-life in *Escherichia coli*, yeast, and mammalian reticulocytes (Gasteiger et al., 2005[33]). 

### Assessment of antigenicity and allergenicity

The antigenic potential of the TgRON proteins was evaluated using two online tools: VaxiJen v2.0 and ANTIGENpro. VaxiJen v2.0 predicts antigenicity based on auto-cross covariance (ACC) transformation of protein sequences, with a default threshold of 0.5 for parasitic organisms and a reported specificity of 70-89 % (Doytchinova and Flower, 2007[20]). ANTIGENpro, on the other hand, is a sequence-based, alignment-free predictor that utilizes a two-stage machine learning framework involving five algorithms and various sequence representations to assess protein antigenicity. The final prediction is generated by a support vector machine (SVM) classifier, which outputs a probability score representing the likelihood of antigenicity. ANTIGENpro was developed using protein microarray data from five different diseases and is capable of predicting antigens in a pathogen-independent manner (Cheng et al., 2005[12]; Magnan et al., 2010[58]). This tool does not support sequences exceeding 1500 amino acids. To assess the allergenic potential of the TgRON proteins, we employed the AlgPred web server, which integrates multiple approaches for allergen prediction, including motif-based and machine learning methods, to improve accuracy. The importance of allergenicity assessment is increasingly recognized due to the growing use of novel proteins in genetically modified foods, biopharmaceuticals, and other applications. The standards established by the Food and Agriculture Organization (FAO) and the World Health Organization (WHO) highlight the need for reliable allergen prediction tools (Saha and Raghava, 2006[68]). Additionally, AllergenFP v1.0 and AllerTOP v2.0 were used to further evaluate the potential allergenicity of the TgRON proteins. Both tools employ descriptor-based fingerprinting methods and machine learning algorithms to distinguish allergens from non-allergens with high accuracy (Dimitrov et al., 2014[18][19]).

### Prediction of subcellular localization, transmembrane domains, signal peptides, and solubility

To predict the subcellular localization of TgRON proteins, the DeepLoc 2.0 web server was utilized. This deep learning-based tool provides multi-label predictions, allowing identification of up to ten possible eukaryotic protein localizations, including the cytoplasm, nucleus, mitochondrion, endoplasmic reticulum, Golgi apparatus, cell membrane, extracellular space, lysosome/vacuole, peroxisome, and chloroplast (Thumuluri et al., 2022[81]). The presence and topology of transmembrane domains in TgRONs were predicted using the DeepTMHMM server (Release 1.0.24), which is capable of detecting both alpha-helical and beta-barrel transmembrane regions (Hallgren et al., 2022[39]). Signal peptides were identified using SignalP 6.0, which predicts signal peptide cleavage sites and distinguishes between signal peptides and transmembrane regions with high accuracy (Teufel et al., 2022[80]).

To assess protein solubility, two servers were employed. The Protein-sol tool estimates solubility based on a single amino acid sequence and compares the result against a solubility database; proteins with scores above 0.45 are considered soluble (Hebditch et al., 2017[41]). Additionally, SOLpro was used to predict the likelihood of protein solubility upon overexpression in *Escherichia coli*. This tool employs a two-stage support vector machine (SVM) model, where initial classifiers analyze various features of the sequence, and a final SVM integrates these predictions to provide a solubility probability score (Cheng et al., 2005[12]; Magnan et al., 2009[57]). It is important to note that SOLpro does not support sequences longer than 1500 amino acids.

### Prediction of post-translational modification sites

To identify potential post-translational modification (PTM) sites in TgRON proteins, several specialized web servers were employed. Phosphorylation sites were predicted using GPS 6.0 (Chen et al., 2023[11]), N-glycosylation sites with NetNGlyc 1.0 (Gupta and Brunak, 2002[38]), and O-glycosylation sites using NetOGlyc 4.0 (Steentoft et al., 2013[77]). Methylation sites were assessed via GPS-MSP 1.0 (Deng et al., 2017[14]), palmitoylation sites with GPS-Palm 1.0 (Ning et al., 2021) (Ning et al., 2021[60]), and acetylation sites through GPS-PAIL 2.0 (Deng et al., 2016[13]). These tools collectively provided insights into the PTM landscape of the TgRON proteins, which may play important roles in protein function, localization, and immune recognition.

### Prediction of secondary and tertiary structures

The secondary structure of TgRON proteins was analyzed using the SOPMA web server, which predicts elements such as alpha helices, beta strands, and random coils based on sequence data (Garnier et al., 1996[32]; Geourjon and Deleage, 1995[34]). For tertiary structure modeling, SWISS-MODEL was used to generate three-dimensional (3D) protein models based on homology with known protein structures (Guex et al., 2009[37]).

### Refinement and validation of TgRON protein 3D models

To improve the quality of the initial 3D structures generated by SWISS-MODEL, the GalaxyRefine web server was employed. This tool enhances template-based models through side-chain repacking followed by molecular dynamics simulation to achieve global structural relaxation and refinement (Heo et al., 2013[42]; Ko et al., 2012[49]; Shin et al., 2014[74]). The accuracy and reliability of the refined 3D models were evaluated using multiple validation tools. The SWISS-MODEL structure assessment module was used to generate Ramachandran plots for visualizing and assessing backbone dihedral angle distributions, helping to verify stereochemical quality (Waterhouse et al., 2024[86]). In addition, the overall structural integrity of each model was analyzed using the ProSA-web server, which evaluates model quality based on Z-score analysis and deviation from experimentally determined structures (Wiederstein and Sippl, 2007[88]).

### Prediction of linear and discontinuous B-cell epitopes

Antigenic epitopes are specific regions on the protein surface that are preferentially recognized by antibodies, making them essential targets in vaccine development and immunodiagnostic applications. B cells play a central role in immune defense by producing antibodies that bind to these epitopes. Accurate prediction of B-cell epitopes not only facilitates vaccine design but also enhances understanding of immune recognition mechanisms. To identify linear B-cell epitopes, the SVMTrip server was utilized with an epitope length set at 20 amino acids (Yao et al., 2012[91]). Additionally, the ABCpred server, which employs artificial neural networks (ANNs) with recurrent connectivity, was used to predict linear epitope regions within antigen sequences. For this tool, a 16-mer epitope length was specified, with a threshold of 0.9 and overlapping filter enabled. ABCpred achieves an average prediction accuracy of 65.93 % (Saha and Raghava, 2006[70], 2007[69]). To further assess the predicted linear epitopes, we evaluated their antigenicity, allergenicity, and solubility using a combination of online tools: VaxiJen v2.0, AllerTOP v. 2.0, AlgPred, AllergenFP v. 1.0, and PepCalc (Dimitrov et al., 2014[18][19]; Doytchinova and Flower, 2007[20]; Saha and Raghava, 2006[68]). For conformational (discontinuous) B-cell epitope prediction, the ElliPro server was employed, which maps epitopes based on the 3D structure of the protein. Default parameters were applied, with a maximum distance threshold of 6 Å and a minimum score of 0.5 (Ponomarenko et al., 2008[63]).

### Prediction of MHC class I and II binding epitopes

To identify cytotoxic T lymphocyte (CTL) epitopes, the NetCTL 1.2 server was used to predict potential MHC class I-binding peptides across 12 supertypes. For broad population coverage, three representative supertypes, A2, A3, and B7, were selected, collectively covering approximately 88.3 % of the global population (Larsen et al., 2007[51]). Subsequently, the immunogenicity of the predicted CTL epitopes was evaluated using the MHC class I immunogenicity prediction tool provided by the Immune Epitope Database (IEDB) (Calis et al., 2013[7]). For the prediction of helper T lymphocyte (HTL) epitopes, the IEDB MHC class II binding prediction tool was employed, using the full human leukocyte antigen (HLA) reference set. This tool reports the lowest percentile rank and the highest binding affinity for each epitope-HLA combination, offering insights into their binding strengths (Vita et al., 2019[82]). To further evaluate the immunological relevance of the predicted epitopes, antigenicity was assessed via VaxiJen v2.0. In addition, the potential of each epitope to induce interleukin-4 (IL-4) and interferon-gamma (IFN-γ) responses was predicted using the IL4pred and IFNepitope servers, respectively (Dhanda et al., 2013[16][17]; Doytchinova and Flower, 2007[20]).

### In silico immune simulation

An immune simulation of a generic protein, represented by its amino acid sequence, was performed using the C-ImmSim server (Rapin et al., 2010[65]). This server models the immune system's response to an antigen through a position-specific scoring matrix (PSSM). It provides insights into various immune parameters, including the production of interferons, cytokines, and vaccine-related antibodies. Additionally, the simulation can assess cellular immunity, specifically helper T-cell type 1 (Th1) and Th2 responses. To simulate the immune response triggered by *T. gondii* RON proteins, a virtual immunological procedure was conducted using this server. The parameters for the simulation were set as follows: random seed 12345, 1050 simulation steps, a simulation volume of 10, and three injections administered at four-week intervals. Time steps were configured to represent real-life intervals of 8 hours, with key time points set at 1, 84, and 168 hours.

## Results

### Physicochemical properties, antigenicity, allergenicity, and solubility

The antigenicity of the examined TgRON proteins was predicted using two servers: VaxiJen v2.0 and ANTIGENpro. Except for TgRON4L1, all proteins were predicted to be probable antigens by VaxiJen v2.0, with antigenicity scores ranging from 0.5007 to 0.8226. The highest score was attributed to TgRON9 (0.8226), while the lowest was recorded for TgRON4L1 (0.4786). These findings were corroborated by ANTIGENpro, which also predicted TgRON9 as the most antigenic protein with a score of 0.9647. Allergenicity predictions were conducted using multiple approaches via the AlgPred server, including MEME/MAST motifs, specific IgE epitope recognition, and a hybrid approach. None of the TgRON proteins displayed allergenic properties. These outcomes were further validated through additional allergenicity prediction tools: AllergenFP 1.0 and AllerTOP v. 2.0, all of which confirmed the non-allergenic nature of the proteins (Table 1(Tab. 1)).

**Figure 2:**
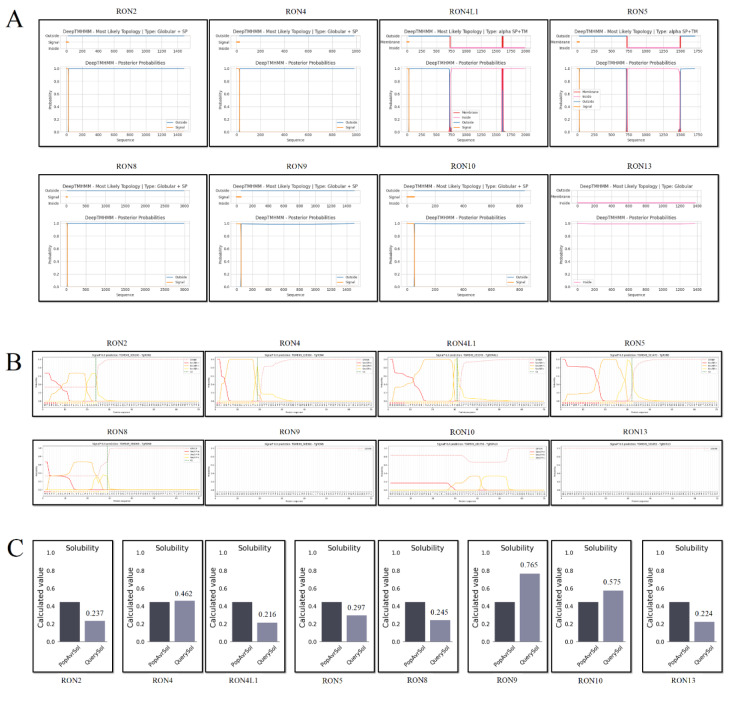
*In silico* characterization of TgRON proteins. (A) Predicted transmembrane domains. (B) Signal peptide prediction results. (C) Solubility predictions for heterologous expression.

Regarding solubility, the SOLpro server predicted that TgRON9 and TgRON10 were soluble, while TgRON2, TgRON4, and TgRON13 were insoluble. Protein-Sol analysis supported these predictions, identifying TgRON4, TgRON9, and TgRON10 as soluble based on their scaled solubility scores of 0.462, 0.765, and 0.575, respectively (Figure 2C(Fig. 2)). The physicochemical analysis revealed that the length of the proteins ranged from 835 amino acids in TgRON10 to 2980 amino acids in TgRON8. Correspondingly, TgRON8 had the highest molecular weight at 328.900 kDa, while TgRON10 had the lowest at 91.370 kDa-both values falling within acceptable ranges for antigenic proteins. The theoretical isoelectric point (pI) values ranged from 4.29 for TgRON9 to 9.61 for TgRON2. The number of negatively charged residues (Asp + Glu) was highest in TgRON8 (314), followed by TgRON9 (295) and TgRON4L1 (221), while TgRON8 also had the highest number of positively charged residues (Arg + Lys) at 329.

All examined proteins had a predicted *in vitro *half-life of 30 hours using mammalian reticulocytes. Instability index values indicated that all proteins were classified as unstable, with scores ranging from 41.74 (TgRON5) to 73.48 (TgRON9). Aliphatic indices varied between 51.17 and 84.63, suggesting moderate to good thermostability. All GRAVY scores were negative, indicating that the proteins are hydrophilic (Table 1(Tab. 1)). The chemical compositions of the proteins were also calculated, with molecular formulas provided for each protein including RON2, RON4, RON4L1, RON5, RON8, RON9, RON10, and RON13.

### Prediction of transmembrane domain, signal peptide, and subcellular localization

The DeepLoc web server was used to predict the subcellular localization of the examined proteins. All proteins were predicted to be extracellular except TgRON9, which was localized to the Golgi apparatus (Table 1(Tab. 1)). Signal peptide analysis using SignalP 6.0 indicated that five proteins, TgRON2, TgRON4, TgRON4L1, TgRON5, and TgRON8, possess potential signal peptides (Supplementary information Table S1; Figure 2B(Fig. 2)). Further analysis revealed the presence of transmembrane regions (TMRs) in two proteins. TgRON4L1 was predicted to contain three TMRs located at positions 718-727, 1596-1605, and 1608-1621, while TgRON5 was predicted to have two at positions 716-725 and 1488-1497 (Figure 2A(Fig. 2)).

### Prediction of PTM sites 

PTM site prediction indicated that all the studied proteins contained N-glycosylation sites. The number of such sites ranged from one in TgRON4L1 and TgRON13 to nine in TgRON9. O-glycosylation was also widespread, with TgRON9 having the highest number of sites (181), and TgRON2 and TgRON13 having the fewest. Palmitoylation sites were predicted in all proteins. Phosphorylation site analysis revealed that TgRON8 had the most sites (144), while TgRON9 had the least (58). Additionally, acetylation site prediction showed TgRON9 and TgRON13 as having the highest (63) and lowest (7) numbers of sites, respectively (Table S2).

### Tertiary and secondary structure prediction

**Figure 3:**
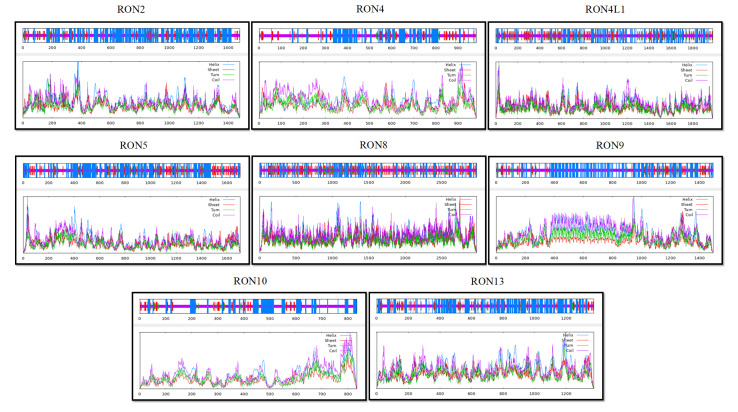
Predicted secondary structure composition of TgRON proteins using the SOPMA server, indicating the distribution of alpha helices, beta strands, turns, and random coils.

**Figure 4:**
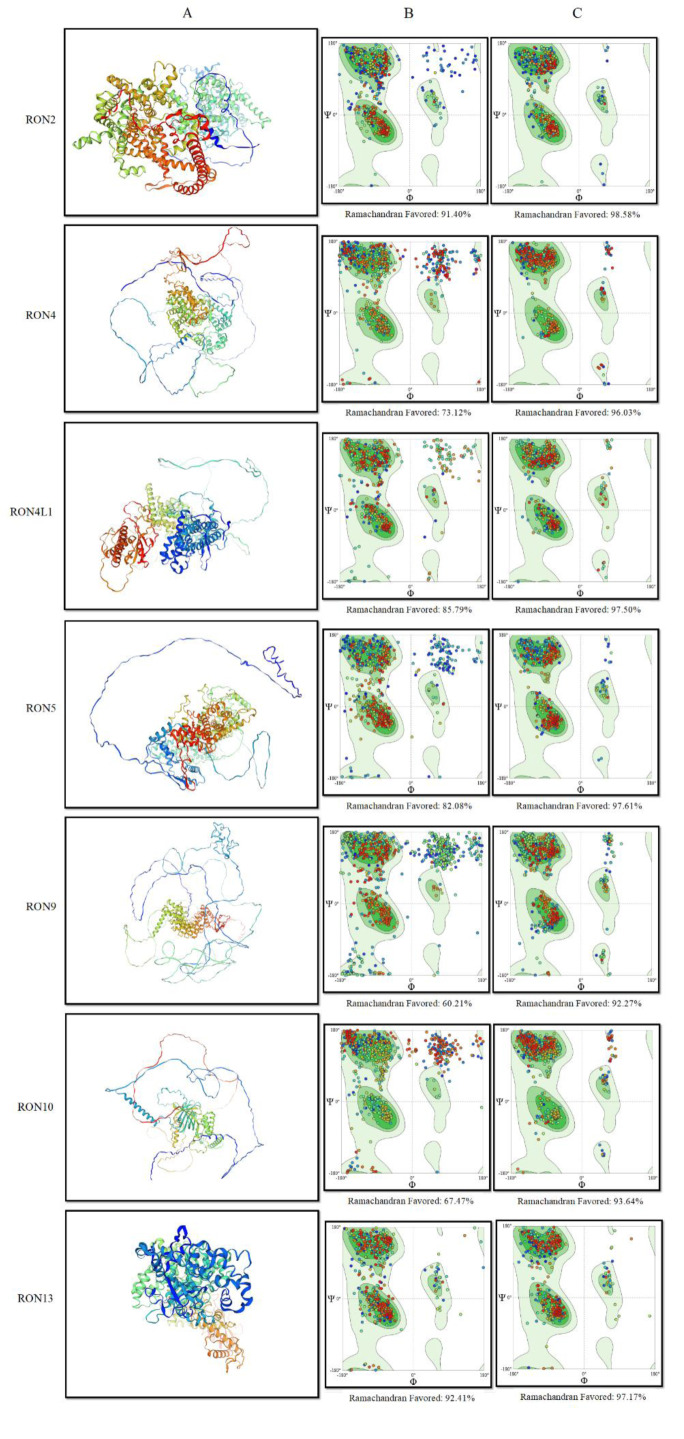
Structural modeling and validation of TgRON Proteins. (A) Predicted three-dimensional structure of TgRON proteins generated using the SWISS-MODEL server. (B) Structural validation of the initial models using quality assessment tools prior to refinement. (C) Validation results following refinement, illustrating improvements in model quality.

Secondary structure analysis using the SOPMA server revealed that TgRON10 had the highest proportion of random coils (57.01 %), followed by TgRON4 (51.52 %) and TgRON9 (46.42 %). The protein with the highest percentage of extended strands was TgRON8 (19.16 %), while TgRON2 had the highest proportion of alpha helices (54.23 %). Conversely, TgRON8 had the lowest percentage of alpha helices (28.36 %), and TgRON9 exhibited the lowest proportion of extended strands (7.30 %) (Figure 3(Fig. 3), Table S3). For tertiary structure prediction, homology modeling of the examined TgRONs was performed using the SWISS-MODEL server. The 3D structures of TgRON2, TgRON4, TgRON4L1, TgRON5, TgRON9, TgRON10, and TgRON13 were modeled based on high-sequence-identity templates. These included B6KV60.1.A (100 %), B6KJ32.1.A (99.80 %), A0A139XSU4.1.A (100 %), A0A086MAK8.1.A (99.83 %), V4ZRL7.1.A (100 %), A0A140CWX2.1.A (99.28 %), and 7nur.1.A (99.46 %) (Figure 4A(Fig. 4)).

### Three-dimensional model validation and refinement

The 3D model refinement was performed using the GalaxyRefine online server, which involves repeated structure perturbation and subsequent structural relaxation via molecular dynamics simulation. For Model 1, structure perturbation was applied only to clusters of side chains, while for Models 2-5, more aggressive perturbations to secondary structure elements and loops were implemented. The triaxial loop closure method was employed to avoid structural breaks caused by the perturbations (Heo et al., 2013[42]; Lee et al., 2016[52]). The refinement process resulted in considerable improvements in the quality of the structures, as assessed by the Ramachandran plot and ProSA-web tools. In the initial crude models, the quality of the structures was as follows: 91.40 %, 73.12 %, 85.79 %, 82.08 %, 60.21 %, 67.47 %, and 92.41 % of the residues for TgRON2, TgRON4, TgRON4L1, TgRON5, TgRON9, TgRON10, and TgRON13, respectively, were positioned in the favored regions (Figure 4B(Fig. 4)). After refinement, these values improved significantly: 98.58 %, 96.03 %, 97.50 %, 97.61 %, 92.27 %, 93.64 %, and 97.17 % of the residues for TgRON2, TgRON4, TgRON4L1, TgRON5, TgRON9, TgRON10, and TgRON13, respectively, were in the favored regions (Figure 4C(Fig. 4)). These improvements suggest that the refinement procedure led to energetically favorable conformations for the protein structures. The Z score, which evaluates the overall quality of the models, also demonstrated that the refinement process enhanced the quality of the 3D structures (Table S4).

### Screening of linear B-cell epitopes

**Figure 5:**
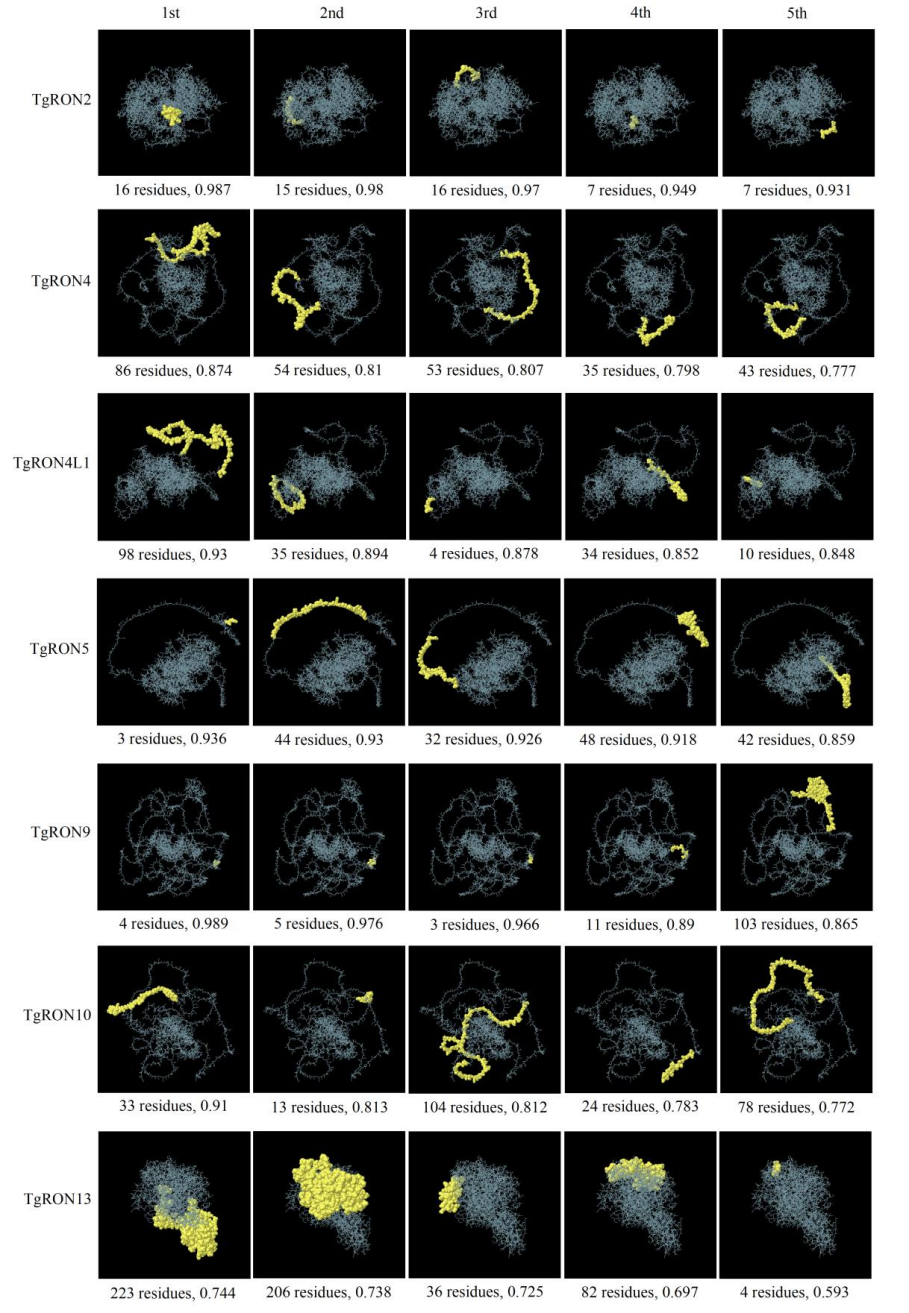
Conformational B-cell epitope mapping on TgRON proteins. Three-dimensional visualization of conformational (discontinuous) B-cell epitopes on TgRON proteins as predicted by the ElliPro server. Epitope lengths and corresponding scores are indicated. TgRON protein structures are shown as white rods, with predicted conformational epitopes are highlighted in yellow.

The linear B-cell epitopes for *T. gondii* proteins were predicted using the ABCpred and SVMTriP servers, followed by allergenicity, antigenicity, and water solubility analyses. From the SVMTriP server, two antigenic, non-allergenic B-cell epitopes with good water solubility were identified: “GLYSEAVRVALRLLRLGHCR” from TgRON4L1 (VaxiJen score: 0.9116) and “LARVSMRHARFVFKAYAMLD” from TgRON13 (VaxiJen score: 0.6880) (Table S5). Additionally, the ABCpred server predicted five antigenic, non-allergenic B-cell epitopes with good water solubility: “QALGIAPPHRGDFENE” from TgRON2 (VaxiJen score: 0.8944), “PGDIKRRLARGEKLPE” from TgRON2 (VaxiJen score: 0.5438), “SAPAQSHETPVAEHAP” from TgRON9 (VaxiJen score: 0.7913), “SQSSETPAEENAQVPK” from TgRON9 (VaxiJen score: 0.8200), and “PTSSSAFRDMVRIADP” from TgRON13 (VaxiJen score: 0.6009) (Table S6). Moreover, conformational B-cell epitopes were predicted using the ElliPro web tool. This tool predicted a range of epitopes for the various TgRON proteins, with 35 epitopes for TgRON2 (scores between 0.987 to 0.512), 17 epitopes for TgRON4 (scores ranging from 0.874 to 0.522), 22 epitopes for TgRON4L1 (scores ranging from 0.93 to 0.502), 27 epitopes for TgRON5 (scores ranging from 0.936 to 0.511), 18 epitopes for TgRON9 (scores ranging from 0.989 to 0.526), 16 epitopes for TgRON10 (scores ranging from 0.91 to 0.502), and 7 epitopes for TgRON13 (scores ranging from 0.744 to 0.542). The five best epitope models with the highest scores are illustrated in Figure 5(Fig. 5).

### CTL and HTL epitope prediction and screening

The prediction and screening of CTL and HTL epitopes were conducted to identify key immunogenic regions involved in limiting both acute and chronic infections. The NetCTL 1.2 server was used to predict CTL epitopes based on MHC supertypes A2, A3, and B7. The results of the immunogenicity screening, including the most immunogenic epitopes, are displayed in Table S7. HTL epitopes were predicted using the IEDB server, which was restricted to the HLA reference set. Three high-ranking epitopes for each protein under study were selected, and the results are presented in Table S8.

### In silico immune simulation

**Figure 6:**
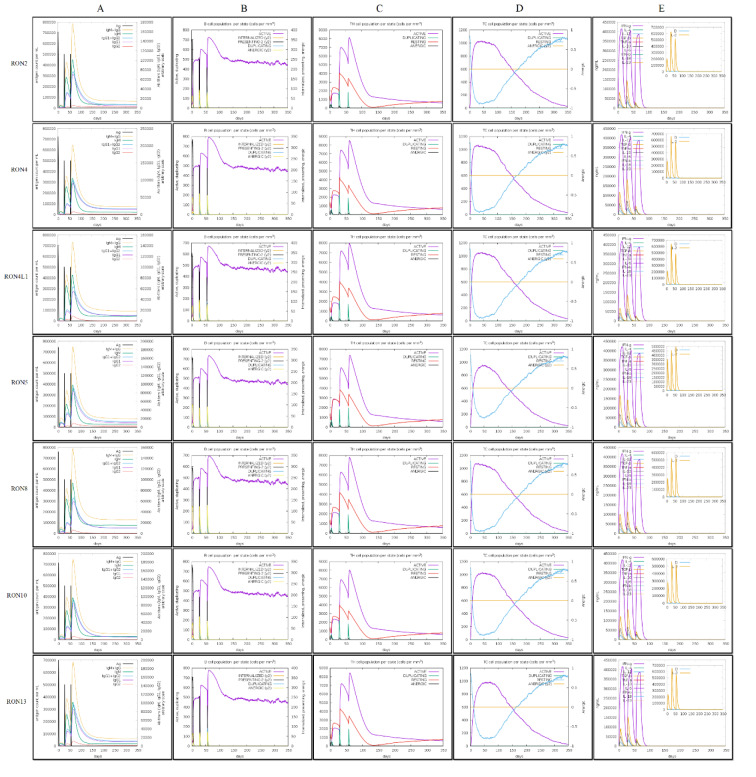
* In silico *simulation of immune responses induced by TgRON proteins. (A) Temporal immunoglobulin production; (B) B-cell population dynamics per functional state (cells/mm^3^); (C) Helper T cell (CD4^+^) population per state (cells/mm^3^); (D) Cytotoxic T cell (CD8^+^) population per state (cells/mm^3^); and (E) Cytokine levels (ng/ml), including key mediators such as IFN-γ.

The *in silico* immune simulation using the CImmSim web server revealed that the injection of all selected proteins significantly enhanced both cell-mediated and humoral immune responses. There was a significant increase in IgM and IgG+IgM titers against the antigenic proteins, as shown in the antibody response graph (Figure 6A(Fig. 6)). A very strong IFN-γ response (up to 400,000 ng/ml) was detected, with a rapid increase following the injection (Figure 6E(Fig. 6)). The peak of the active B-cell populations for each protein reached approximately: ~720 cells/mm³ for TgRON2, ~800 cells/mm³ for TgRON4, ~680 cells/mm³ for TgRON4L1, ~720 cells/mm³ for TgRON5, ~700 cells/mm³ for TgRON8, ~750 cells/mm³ for TgRON10, and ~780 cells/mm³ for TgRON13 (Figure 6B(Fig. 6)). Additionally, the peak of the active TH cell populations reached approximately: ~8000 cells/mm³ for TgRON2, ~8600 cells/mm³ for TgRON4, ~8000 cells/mm³ for TgRON4L1, ~7400 cells/mm³ for TgRON5, ~7800 cells/mm³ for TgRON8, ~8000 cells/mm³ for TgRON10, and ~8500 cells/mm³ for TgRON13 (Figure 6C(Fig. 6)). The TC cell populations also increased by approximately: ~1000 cells/mm³ for TgRON2, ~1040 cells/mm³ for TgRON4, ~1030 cells/mm³ for TgRON4L1, ~950 cells/mm³ for TgRON5, ~1050 cells/mm³ for TgRON8, ~1010 cells/mm³ for TgRON10, and ~990 cells/mm³ for TgRON13 (Figure 6D(Fig. 6)). Significant percentages of memory B and T cells were also elicited following the injection of the selected TgRONs, highlighting their potential as candidates for immunization. Further details are provided in Figure 6(Fig. 6).

## Discussion

This study aimed to identify promising vaccine targets against *T. gondii*, a globally significant zoonotic parasite, through comprehensive *in silico *analyses. With computational methods playing an increasingly vital role in epitope-based vaccine development, we focused on evaluating several rhoptry neck (RON) proteins based on their immunogenicity and biochemical characteristics. Among the proteins analyzed, TgRON9 was identified as the most promising candidate, exhibiting high predicted antigenicity, favorable solubility, and non-allergenic properties. These features support its potential as a viable target for subsequent experimental validation in the context of toxoplasmosis vaccine development.

All analyzed TgRON proteins were found to be non-allergenic, reinforcing their safety profile as potential immunogens. TgRON9 and TgRON10 also demonstrated predicted solubility, which facilitates downstream processes such as expression and purification. The high molecular weight of TgRON8 and other RON proteins, exceeding 10 kDa, aligns with established findings that such proteins are generally more immunogenic (Berzofsky, 1993[5]). Physicochemical analyses revealed that all proteins exhibited instability indices, suggesting potential expression challenges. However, the favorable aliphatic indices and hydrophilic nature of the proteins suggest acceptable thermal stability and solubility, supporting their suitability in biological systems. The calculated isoelectric points and charge profiles provide valuable guidance for optimizing buffer systems and expression conditions in future experimental setups (Xia, 2007[89]). These favorable physicochemical properties are important for future selection of expression vectors and purification experiments (Dey et al., 2014[15]).

Subcellular localization predictions indicated that most RON proteins, except TgRON9, are secreted extracellularly, a feature that enhances their accessibility to the host immune system (Gillani and Pollastri, 2024[35]). The presence of signal peptides in five of the proteins supports their secretory nature, while the identification of transmembrane domains in TgRON4L1 and TgRON5 may hint at functional roles related to host membrane interaction, which could influence their structural presentation and immunogenic potential (Fink et al., 2012[24]; Owji et al., 2018[61]).

PTM predictions further improved the understanding of the RON proteins' regulatory complexity. Glycosylation, phosphorylation, acetylation, and palmitoylation all represent crucial regulatory mechanisms that influence protein behavior (Li et al., 2025[54]; Ramazi and Zahiri, 2021[64]). The abundance of N- and O-glycosylation sites, particularly in TgRON9, suggests potential roles in protein stability and immune recognition, as glycosylation can shield epitopes or modulate their immunogenicity (Karve and Cheema, 2011[45]; Ramazi and Zahiri, 2021[64]). This aligns with existing literature that emphasizes glycosylation's significance in pathogen-host interactions and immune evasion strategies (Lin et al., 2020[55]; Sperandio et al., 2009[76]). Similarly, phosphorylation and acetylation patterns, especially the high phosphorylation count in TgRON8 and acetylation in TgRON9, point to these proteins' involvement in dynamic cellular signaling and structural modifications (Li et al., 2025[54]; Ramazi and Zahiri, 2021[64]). These PTMs can alter protein conformation, localization, and interaction networks, ultimately influencing their immunogenic profile (Lee et al., 2009[53]; Ramazi and Zahiri, 2021[64]). 

The predominance of random coils, particularly in TgRON10, and the varied proportions of alpha helices and extended strands across different TgRON proteins may influence protein flexibility and antigenic exposure. Regions with random coils are generally more accessible to immune surveillance, potentially enhancing antigenicity (Berjanskii and Wishart, 2008[4]). In contrast, alpha helices and beta strands may contribute to protein stability and specific epitope configurations. Previous studies have noted that structural elements such as beta turns and alpha helices can maintain the protein's tertiary integrity and promote effective antibody interactions (Foroutan et al., 2018[29]; Yada et al., 1988[90]).

Tertiary structure modeling confirmed high-confidence homology with known templates, underscoring the structural conservation among the RON proteins analyzed. Since the 3D structure of a protein is closely tied to its biological function, insights into these spatial configurations are vital for understanding functional roles in host-pathogen interactions and for rational vaccine design (Foroutan et al., 2018[29]; Schueler-Furman et al., 2005[73]; Wang et al., 2016[84]). The refinement of the 3D structures using GalaxyRefine significantly improved the model quality, as evidenced by the Ramachandran plot, ProSA-web tool results, and Z scores. The enhancement in the percentage of residues located in the favored regions of the Ramachandran plot suggests a more stable and energetically favorable conformation for the refined models (Carrascoza et al., 2014[9]; Carugo and Djinovic-Carugo, 2013[10]). The use of structure perturbation and molecular dynamics relaxation, particularly with the triaxial loop closure method, contributed to this structural improvement, ensuring that the refined models maintain physical realism without introducing breaks or distortions (Heo et al., 2013[42]; Lee et al., 2016[52]). Additionally, the Z scores provided a further quantitative measure of the refinement's success, confirming the increase in the structural quality of the proteins (Benkert et al., 2011[3]). The consistency in improvements across various models, such as TgRON2, TgRON4, and others, highlights the robustness of the refinement procedure. These results indicate that the GalaxyRefine server is an effective tool for refining protein structures, providing a more accurate and reliable model for further computational studies or experimental validation.

The identification of linear B-cell epitopes is crucial for the development of effective antibodies that can restrict parasite adherence and promote their removal via opsonization by immune cells such as macrophages (Khan and Moretto, 2022[47]; Sayles et al., 2000[72]). In this study, the use of two distinct servers-ABCpred and SVMTriP-enabled the prediction of multiple linear B-cell epitopes from TgRON proteins with high antigenicity, non-allergenicity, and favorable solubility characteristics. The selection of epitopes with high VaxiJen scores indicates their potential for inducing a strong immune response (Doytchinova and Flower, 2007[20]). The epitopes identified in the SVMTriP server, particularly those from TgRON4L1 and TgRON13, show promising antigenicity, which suggests that these epitopes could be targeted in vaccine development. The additional epitopes identified through the ABCpred server further highlight the diversity of potential targets for B-cell immune responses.

Furthermore, the use of the ElliPro web tool to predict conformational B-cell epitopes adds another layer of information, emphasizing the importance of protein conformation in antigen-antibody interactions (Ponomarenko et al., 2008[63]). The predicted epitopes in TgRON proteins, particularly those with higher scores, could be key to understanding how these proteins interact with immune system components (Kazi et al., 2018[46]; Yurina and Adianingsih, 2022[93]). The large number of epitopes predicted for each protein underscores the potential for these targets in immunological studies and vaccine design (Foroutan et al., 2025[30]; Yurina and Adianingsih, 2022[93]). These findings collectively contribute to the understanding of how specific epitopes in TgRON proteins may play a role in inducing protective immunity against *T. gondii* infection, offering valuable insights for future therapeutic and vaccine development strategies. 

The generation of IFN-γ by T cells plays a critical role in limiting both acute and chronic *T. gondii* infection, as it prevents the reactivation of tissue cysts and controls the acute phase of the infection (El-Kady, 2011[22]; Khan and Moretto, 2022[47]). The function of both T CD4^+^ and T CD8^+ ^cells is essential in managing the infection (Khan et al., 2019[48]). The prediction of CTL epitopes using the NetCTL 1.2 server and HTL epitopes through the IEDB server provides valuable insights into potential targets for immune responses. The immunogenicity screening results, including the identification of the most immunogenic epitopes, and the high-ranking HTL epitopes offer important candidates for further exploration in vaccine development and therapeutic interventions aimed at enhancing immune responses against *T. gondii*.

The *in silico* immune simulation results demonstrated that the selected TgRON proteins effectively elicited robust immune responses, enhancing both the humoral and cell-mediated immunity. The marked increase in IgM and IgG+IgM titers suggests a strong antibody response, which is essential for limiting infection and promoting immunity (Sayles et al., 2000[72]). The significant elevation in IFN-γ levels, which is a key cytokine in controlling *T. gondii* infection, further supports the potential of these proteins in inducing protective immunity (El-Kady, 2011[22]; Khan and Moretto, 2022[47]). The activation of B-cells, TH cells, and TC cells, along with the generation of memory B and T cells, underscores the ability of the selected proteins to stimulate a comprehensive immune response, which is crucial for long-term immunity. Although our study relied on *in silico* methods, previous research supports the potential of RON proteins to enhance immune responses. For example, Zhao et al. (2016[97]) demonstrated that a DNA vaccine encoding *T. gondii* RON5p elicited humoral and cellular immune responses, prolonged survival in mice, and reduced the brain cyst burden. The concordance between our *in silico* findings and these experimental results suggests that RON proteins may serve as promising candidates for immunization.

*In silico* techniques offer a powerful and cost-effective means of rapidly identifying potential antigenic proteins, including those not yet experimentally expressed (Flower et al., 2010[25]; Romano et al., 2011[67]). As these computational tools evolve, their utility in pre-screening candidates for vaccine development continues to grow. These methods enable researchers to narrow down targets efficiently, thereby saving time and resources for downstream laboratory validation. However, despite these advantages, *in silico* approaches are not without limitations. Their predictive accuracy is inherently constrained by the quality and completeness of reference datasets, algorithmic assumptions, and the lack of biological context (Can et al., 2020[8]; Flower et al., 2010[25]; Kazi et al., 2018[46]). Variability in predictions, such as antigenicity, allergenicity, solubility, and immunogenicity, can result from differences between computational tools. For example, TgRON4 was predicted to be both soluble and insoluble depending on whether the Protein-Sol or SOLpro server was used. Similarly, immune simulation platforms often fall short in replicating the full complexity of host responses observed *in vivo*. These discrepancies highlight the importance of viewing *in silico* results as hypothesis-generating rather than conclusive. While our simulations align with some previously reported experimental data (Zhao et al., 2016[97]), we emphasize that laboratory validation remains essential. Integrating computational predictions with robust wet-lab experiments strengthens the reliability of vaccine design pipelines and enhances the likelihood of identifying truly protective antigens (Foroutan et al., 2020[28]; Goodswen et al., 2023[36]; Saldanha et al., 2023[71]).

## Conclusion

This study provides a comprehensive *in silico* evaluation of several *T. gondii* RON proteins, highlighting their structural features, subcellular localization, and immunogenic potential. The identification of promising CTL, HTL, and B-cell epitopes, particularly within TgRON9, offers a valuable starting point for the development of novel vaccine candidates against toxoplasmosis. Our findings emphasize the utility of computational approaches in narrowing down viable antigen targets, thereby reducing time and cost associated with experimental trial-and-error. However, the predictive nature of *in silico* methods necessitates subsequent experimental validation to confirm immunogenicity and protective efficacy. Future work should focus on validating these findings through laboratory studies, optimizing antigen delivery platforms, and evaluating adjuvant combinations to enhance immune responses. These results highlight the role of immunoinformatics as a vital component in the early phases of vaccine design, with its full potential realized when coupled with empirical testing.

## Notes

Masoud Foroutan and Hany M. Elsheikha (School of Veterinary Medicine and Science, Faculty of Medicine and Health Sciences, University of Nottingham, Sutton Bonington Campus, Loughborough, LE12 5RD, UK; Tel: +44 01159516445, E-mail: Hany.Elsheikha@nottingham.ac.uk) contributed equally as corresponding author.

Masoud Foroutan and Amir Karimipour-Saryazdi contributed equally to this work.

## Declaration

### Data availability statement

The data that support the findings of this study are available from the corresponding author upon reasonable request.

### Authors' contributions

**Conceptualization:** M. Foroutan; **Methodology: **M. Foroutan, F. Ghaffarifar, and A. Karimipour-Saryazdi; **Software:** M. Foroutan and A. Dalir Ghaffari; **Validation: **M. Foroutan, H. Elsheikha, A. Dalir Ghaffari, H. Majidiani, and A. Karimipour-Saryazdi; **Formal analysis:** M. Foroutan and A. Dalir Ghaffari; **Investigation:** M. Foroutan, A. Dalir Ghaffari, A. Karimipour-Saryazdi, and H. Majidiani; **Resources:** M. Foroutan and A. Dalir Ghaffari; **Data Curation:** M. Foroutan, A. Karimipour-Saryazdi, and A. Dalir Ghaffari; **Writing - original draft preparation:** M. Foroutan, A. Dalir Ghaffari, A. Karimipour-Saryazdi, and H. Majidiani; **Writing - review and editing: **H. Elsheikha and F. Ghaffarifar; **Visualization:** M. Foroutan and A. Dalir Ghaffari; **Supervision:** M. Foroutan; **Project administration:** M. Foroutan; and **Funding acquisition:** M. Foroutan. All authors read and approved the final version of the manuscript.

### Funding

This study was supported by a grant from Abadan University of Medical Sciences, Abadan, Iran (Award No. 1835).

### Ethics approval and consent to participate

This study received approval from the Abadan University of Medical Sciences Research Ethics Committee (Approval No. IR.ABADANUMS.REC.1403.010).

### Conflict of interest 

The authors declare no competing financial interests or personal relationships that could have influenced the work reported in this paper.

### Use of artificial intelligence

The authors declare that they used no artificial intelligence software for preparing figure, tables, and texts.

## Supplementary Material

Supplementary information

## Figures and Tables

**Table 1:**
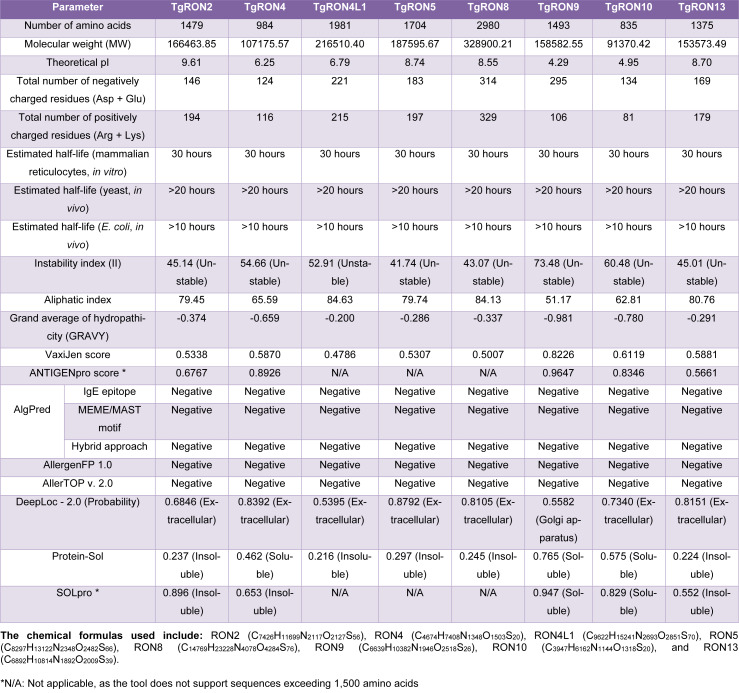
Prediction of the physicochemical properties, antigenicity, solubility, allergenicity, and subcellular localization of TgRONs

**Figure 1:**
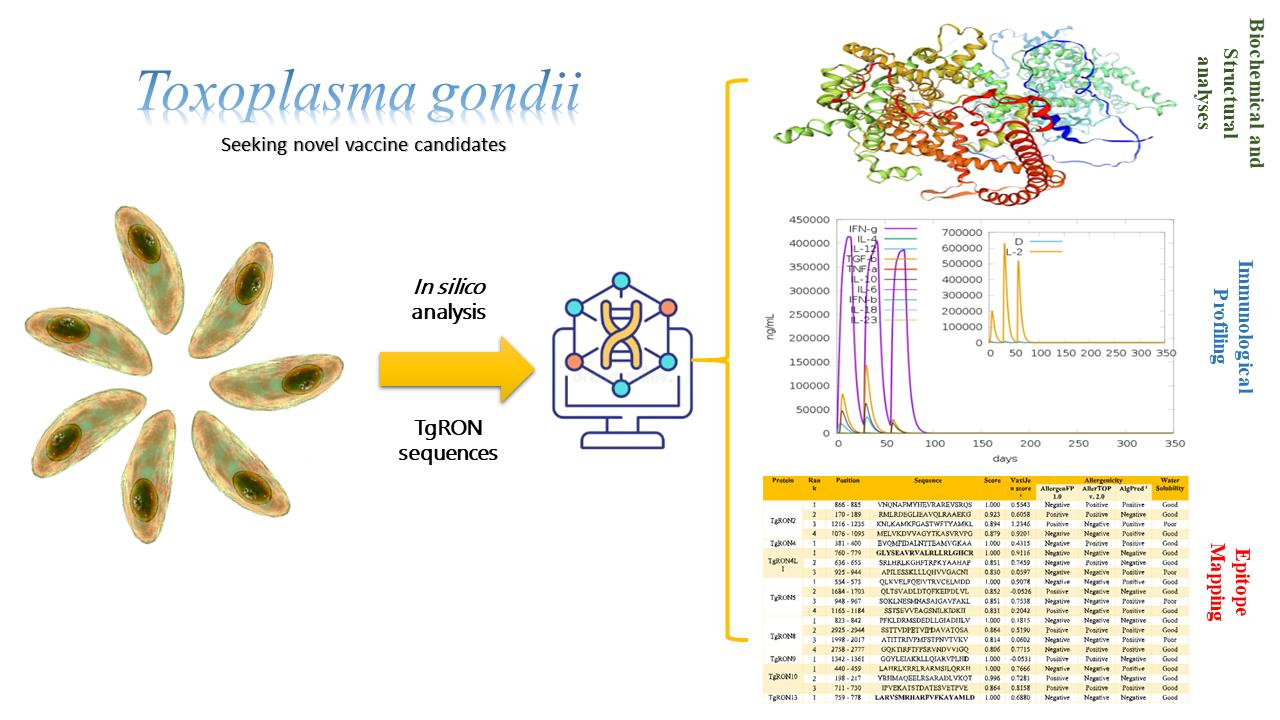
Graphical abstract
