# Supplementary material for: In silico evaluation of Toxoplasma gondii rhoptry neck proteins (TgRONs) for potential immunogenic epitopes
Source: EXCLI J. 2025 Jul 10;24:749–73. doi: 10.17179/excli2025-8304 (PMC12381366; doi:10.17179/excli2025-8304)
Supplement: Supplementary information [file EXCLI-24-749-s-001.pdf]

EXCLI Journal 2025;24:749-773 – ISSN 1611-2156
Received: February 24, 2025, accepted: June 25, 2025, published: July 10, 2025

Supplementary information to:

Original article:

IN SILICO EVALUATION OF *TOXOPLASMA GONDII* RHOPTRY NECK PROTEINS (TGRONS) FOR POTENTIAL IMMUNOGENIC EPITOPES

Masoud Foroutan[1]*#, Hany M. Elsheikha[2]*, Amir Karimipour-Saryazdi[3]#, Ali Dalir Ghaffari[4], Fatemeh Ghaffarifar[3], Hamidreza Majidiani[5,6]

1 Department of Basic Medical Sciences, Faculty of Medicine, Abadan University of Medical Sciences, Abadan, Iran
2 School of Veterinary Medicine and Science, Faculty of Medicine and Health Sciences, University of Nottingham, Loughborough, LE12 5RD, UK
3 Department of Parasitology, Faculty of Medical Sciences, Tarbiat Modares University, Tehran, Iran
4 Department of Parasitology and Mycology, Faculty of Medicine, Shahed University, Tehran, Iran
5 Healthy Aging Research Centre, Neyshabur University of Medical Sciences, Neyshabur, Iran
6 Department of Basic Medical Sciences, Neyshabur University of Medical Sciences, Neyshabur, Iran

\# These authors contributed equally to this work.

* **Corresponding authors:** Masoud Foroutan (Ph.D.; Assistant Professor), Department of Basic Medical Sciences, Faculty of Medicine, Abadan University of Medical Sciences, Abadan, Iran; P.O. Box: 6313833177; Tel: +98-61-53265361;
E-mail: masoud_foroutan_rad@yahoo.com
Hany M. Elsheikha (Ph.D.; Professor), School of Veterinary Medicine and Science, Faculty of Medicine and Health Sciences, University of Nottingham, Sutton Bonington Campus, Loughborough, LE12 5RD, UK; Tel: +44 01159516445;
E-mail: Hany.Elsheikha@nottingham.ac.uk

https://dx.doi.org/10.17179/excli2025-8304

This is an Open Access article distributed under the terms of the Creative Commons Attribution License (https://creativecommons.org/licenses/by/4.0/).

EXCLI Journal 2025;24:749-773 – ISSN 1611-2156
Received: February 24, 2025, accepted: June 25, 2025, published: July 10, 2025

Table S1: Prediction of signal peptides of eight TgRON proteins based on *in silico* analysis.

Protein name	Signal peptide (Sec/SPI)		Cleavage between pos.	Probability
	**Yes/No**	**Likelihood**		
TgRON2	Yes	0.6666	24 and 25	0.6502
TgRON4	Yes	1	19 and 20	0.8381
TgRON4L1	Yes	0.9999	31 and 32	0.9929
TgRON5	Yes	0.9997	32 and 33	0.7926
TgRON8	Yes	0.6666	29 and 30	0.3259
TgRON9	No	0	-	-
TgRON10	No	0.2511	-	-
TgRON13	No	0	-	-

EXCLI Journal 2025;24:749-773 – ISSN 1611-2156
Received: February 24, 2025, accepted: June 25, 2025, published: July 10, 2025

Table S2: Prediction of post-translational modification (PTM) sites in TgRON proteins, including glycosylation, methylation, palmitoylation, phosphorylation and acetylation regions.

Proteins	No. of O-glycosylation sites [1]	No. of N-glycosylation sites [2]	No. of methylation sites [3]	No. of palmitoylation sites [4]	No. of phosphorylation sites [5]	No. of acetylation sites [6]
**TgRON2**	37	5	3	4	90	11
**TgRON4**	90	3	0	3	97	15
**TgRON4L1**	63	1	4	8	95	9
**TgRON5**	48	5	4	4	90	10
**TgRON8**	42	5	2	7	144	14
**TgRON9**	181	9	1	6	58	63
**TgRON10**	67	3	1	3	59	14
**TgRON13**	26	1	1	7	74	7

[1] NetOGlyc 4.0 (Prediction of O-glycosylation sites)
[2] NetNGlyc 1.0 (Prediction of N-glycosylation sites)
[3] GPS-MSP 1.0 (Prediction of methylation sites)
[4] GPS-Palm 1.0 (Prediction of palmitoylation sites)
[5] GPS 6.0 (Prediction of phosphorylation sites)
[6] GPS-PAIL 2.0 (Prediction of acetylation on internal lysines)

EXCLI Journal 2025;24:749-773 – ISSN 1611-2156
Received: February 24, 2025, accepted: June 25, 2025, published: July 10, 2025

Table S3: Secondary structure prediction of TgRON proteins using the SOPMA method, showing the distribution of helices, strands, coils, and turns.

Parameter n (%)	RON2	RON4	RON4L1	RON5	RON8	RON9	RON10	RON13
Alpha helix	802 (54.23)	318 (32.32)	683 (34.48)	784 (46.01)	845 (28.36)	620 (41.53)	249 (29.82)	683 (49.67)
Extended strand	144 (9.74)	105 (10.67)	316 (15.95)	253 (14.85)	571 (19.16)	109 (7.30)	78 (9.34)	135 (9.82)
Beta turn	68 (4.60)	54 (5.49)	156 (7.87)	104 (6.10)	199 (6.68)	71 (4.76)	32 (3.83)	52 (3.78)
Random coil	465 (31.44)	507 (51.52)	826 (41.70)	563 (33.04)	1365 (45.81)	693 (46.42)	476 (57.01)	505 (36.73)
Sequence length	1479	984	1981	1704	2980	1493	835	1375

EXCLI Journal 2025;24:749-773 – ISSN 1611-2156
Received: February 24, 2025, accepted: June 25, 2025, published: July 10, 2025

Table S4: Overall quality assessment of 3D models of the TgRON proteins using ProSA-web Z-scores.

Proteins	Z Score (before refinement)	Z Score (after refinement)
TgRON2	-12.57	-13.15
TgRON4	-9.05	-9.51
TgRON4L1	-13.92	-14.29
TgRON5	-9.51	-10.65
TgRON9	-3.72	-4.32
TgRON10	-5.80	-6.26
TgRON13	-15.54	-15.96

EXCLI Journal 2025;24:749-773 – ISSN 1611-2156
Received: February 24, 2025, accepted: June 25, 2025, published: July 10, 2025

Table S5: Predicted linear B-cell epitopes of the TgRON proteins using the SVMTriP server, including epitope length and antigenicity scores.

Protein	Rank	Position	Sequence	Score	VaxiJen score [1]	Allergenicity			Water Solubility
						AllergenFP 1.0	AllerTOP v. 2.0	AlgPred [2]	
TgRON2	1	866 - 885	VNQNAFMYHEVRAREVSRQS	1.000	0.5543	Negative	Positive	Positive	Good
	2	170 - 189	RMLRDEGLIEAVQLRAAEKG	0.923	0.6058	Positive	Positive	Negative	Good
	3	1216 - 1235	KNLKAMKFGASTWFTYAMKL	0.894	1.2346	Positive	Negative	Positive	Poor
	4	1076 - 1095	MELVKDVVAGYTKASVRVPG	0.879	0.9201	Negative	Negative	Positive	Good
TgRON4	1	381 - 400	EVQMFIDALNTTEAMVGKAA	1.000	0.4315	Negative	Positive	Positive	Good
TgRON4L1	1	760 - 779	**GLYSEAVRVALRLLRLGHCR**	1.000	0.9116	Negative	Negative	Negative	Good
	2	636 - 655	SRLHRLKGHFTRPKYAAHAP	0.851	0.7459	Negative	Positive	Negative	Good
	3	925 - 944	APILESSKLLLQHVVGACNI	0.830	0.0597	Negative	Negative	Positive	Poor
TgRON5	1	554 - 573	QLKVELFQEIVTRVCELMDD	1.000	0.9078	Negative	Negative	Positive	Good
	2	1684 - 1703	QLTSVADLDTQFKEIPDLVL	0.852	-0.0526	Positive	Negative	Negative	Good
	3	948 - 967	SQKLNESMNASAIGAVFAKL	0.851	0.7538	Negative	Negative	Positive	Poor
	4	1165 - 1184	SSTSEVVEAGSNILKIDKII	0.831	0.2042	Positive	Negative	Positive	Good
TgRON8	1	823 - 842	PFKLDRMSDEDLLGIADHLV	1.000	0.1815	Negative	Negative	Negative	Good
	2	2925 - 2944	SSTTVDPETVIPDAVATQSA	0.864	0.5190	Positive	Positive	Positive	Good
	3	1998 - 2017	ATITTRIVPMFSTPNVTVKV	0.814	0.0602	Negative	Negative	Positive	Poor
	4	2758 - 2777	GQKTIRFTFPSRVNDVVIGQ	0.806	0.7715	Negative	Positive	Positive	Good
TgRON9	1	1342 - 1361	GGYLEIAKRLLQIARVPLHD	1.000	-0.0531	Positive	Positive	Negative	Good
TgRON10	1	440 - 459	LAHRLKRRLRARMSILQRKH	1.000	0.7666	Negative	Negative	Positive	Good
	2	198 - 217	YRHMAQEELRSARADLVKQT	0.996	0.7281	Positive	Negative	Negative	Good
	3	711 - 730	IPVEKATSTDATESVETPVE	0.864	0.8158	Positive	Positive	Positive	Good
TgRON13	1	759 - 778	**LARVSMRHARFVFKAYAMLD**	1.000	0.6880	Negative	Negative	Negative	Good

[1] VaxiJen score: Threshold for parasites: 0.5
[2] AlgPred: A hybrid approach (SVMc + IgE epitope + ARPs BLAST + MAST) was applied.

EXCLI Journal 2025;24:749-773 – ISSN 1611-2156
Received: February 24, 2025, accepted: June 25, 2025, published: July 10, 2025

Table S6: Linear B-cell epitopes of TgRON proteins predicted by ABCpred, with the corresponding scores and positions.

Protein	Rank	Sequence	Start position	Score	VaxiJen Score [1]	Allergenicity			Water Solubility
						AllergenFP 1.0	AllerTOP v. 2.0	AlgPred [2]	
TgRON2	1	HGSWSWSGTPPEVQTT	127	0.96	0.3166	Negative	Positive	Positive	Poor
	2	EQETKQPPRPRNLHNP	1424	0.95	0.3621	Negative	Negative	Positive	Good
	3	**QALGIAPPHRGDFENE**	358	0.94	0.8944	Negative	Negative	Negative	Good
	4	DEMCDNHRGPKRRGQK	452	0.93	0.6788	Positive	Negative	Positive	Good
	5	**PGDIKRRLARGEKLPE**	916	0.92	0.5438	Negative	Negative	Negative	Good
	5	SVLFTDAAEPDSDATP	40	0.92	0.9815	Negative	Positive	Positive	Good
	6	HKLSQGRNLPRSQRSL	735	0.91	0.1575	Negative	Positive	Positive	Good
	6	NERSEYAVWFGVKVDM	1030	0.91	1.0455	Positive	Negative	Positive	Good
	7	GIPIPNLTNWDAQLNS	937	0.90	0.7627	Positive	Negative	Positive	Poor
	7	RGLVRETDDMIKRWAE	897	0.90	-0.1386	Negative	Positive	Negative	Good
	7	YQTGGARKHKNRDMLP	470	0.90	0.4717	Positive	Negative	Negative	Good
	7	PSSWGDTELDSLGVPP	1439	0.90	0.6182	Positive	Negative	Positive	Good
	7	PQAIAKATTSAARVAT	1325	0.90	0.2573	Negative	Positive	Positive	Poor
TgRON4	1	TPEIPAKSEEDSEAAE	455	0.96	0.8905	Positive	Positive	Positive	Good
	1	TTVQSSPPTPAPRMYP	22	0.96	0.7231	Negative	Negative	Positive	Poor
	2	HTLDFDAVSPRKNKNK	944	0.95	0.3832	Negative	Negative	Positive	Good
	2	AESRLTPGTYRSELHI	46	0.95	1.0976	Positive	Negative	Negative	Good
	3	GGTSEGPQVPQSGIPP	197	0.94	0.7686	Positive	Positive	Positive	Good
	3	EGTSESPPVPQLGTPP	100	0.94	0.1613	Negative	Negative	Positive	Good
	4	AESSDEDPLPAENATA	913	0.92	0.6053	Negative	Positive	Positive	Good
	4	HALSQAICDPNISAQY	770	0.92	0.0614	Positive	Positive	Positive	Poor
	4	ERPIGRHALSQAICDP	764	0.92	0.1295	Positive	Positive	Negative	Good
	4	ISKAHGPLTRVPEWTP	509	0.92	-0.3404	Negative	Positive	Negative	Good

EXCLI Journal 2025;24:749-773 – ISSN 1611-2156
Received: February 24, 2025, accepted: June 25, 2025, published: July 10, 2025

	5	ALILEDPGTPKAHAQL	595	0.91	0.4206	Positive	Negative	Positive	Good
	5	TPAPRMYPNMNERPLS	30	0.91	0.4026	Positive	Negative	Positive	Good
	5	ASKGIYPNLDELRQTQ	295	0.91	-0.0254	Positive	Positive	Positive	Good
	6	SRRVIHPVRHRSRTAP	889	0.90	0.4376	Negative	Negative	Positive	Good
TgRON4L1	1	DPEISEGCRMDSVKST	1390	0.96	0.5336	Positive	Positive	Negative	Good
	2	GVLSVLPGTLLFECDP	84	0.92	0.7886	Negative	Negative	Negative	Poor
	2	TTMPTPGTWMKWNDDT	824	0.92	0.8111	Negative	Positive	Positive	Poor
	3	TFLISLPPDSNNGIYR	839	0.91	0.5422	Negative	Negative	Positive	Poor
	4	DPTSREDATMRKLVAY	98	0.90	0.1257	Negative	Negative	Negative	Good
	4	ARRIDDMVFFDLHDIQ	805	0.90	-0.2903	Negative	Positive	Positive	Good
	4	ATTVTMPPRVKAPPVP	33	0.90	0.3325	Negative	Positive	Positive	Poor
TgRON5	1	AQEGDRPPPYNPDADE	360	0.97	0.4070	Negative	Negative	Positive	Good
	2	QGAGARPPFFRGGVDP	150	0.96	-0.0921	Negative	Negative	Positive	Good
	3	RQHGSGPPRPAPRRAA	89	0.93	0.5211	Negative	Negative	Positive	Good
	3	TVGPADYDEERPEQTP	176	0.93	0.8374	Negative	Positive	Negative	Good
	4	ATGGQSSRPPNPTVSP	281	0.92	0.8633	Positive	Positive	Positive	Good
	4	EAEADDERKEDSEDNT	1264	0.92	1.5514	Positive	Negative	Positive	Good
	5	CELMDDPESFLKTVPI	568	0.91	0.6877	Negative	Negative	Positive	Good
	5	TEQASATTEDTKIASA	329	0.91	1.3527	Negative	Positive	Positive	Good
	5	HEIVQSRTVGPADYDE	169	0.91	0.4313	Negative	Negative	Positive	Good
	5	GMGMWTGKVFSTHLTF	1493	0.91	1.2795	Negative	Negative	Positive	Poor
	5	AVQTDAQPLPKAVQTD	1309	0.91	0.6035	Positive	Negative	Positive	Good
	6	HQSGFMKLQRHGNDNS	722	0.90	0.3752	Negative	Positive	Positive	Good
TgRON9	1	**SAPAQSHETPVAEHAP**	306	0.96	0.7913	Negative	Negative	Negative	Good
	2	HEEGQETEQGSEEANS	287	0.95	1.5336	Positive	Positive	Positive	Good
	2	LEKHADPVARDAHGNT	1319	0.95	0.8452	Negative	Positive	Negative	Good
	3	KGGEGASTTQHEEGQE	277	0.93	1.3683	Positive	Negative	Positive	Good

	3	FRAQRWQTPHNNRRTP	117	0.93	0.9576	Positive	Positive	Positive	Good
	4	NRMGMRPLHYVARYPT	1145	0.92	-0.2445	Negative	Positive	Positive	Poor
	5	QSPEQAQGQERENASQ	840	0.91	0.8059	Positive	Negative	Positive	Good
	5	KSGGKTGTDGHEKTNE	256	0.91	1.8938	Positive	Positive	Positive	Good
	6	EQPAETQEGSDAEADA	883	0.90	1.3980	Negative	Negative	Positive	Good
	6	**SQSSETPAEENAQVPK**	710	0.90	0.8200	Negative	Negative	Negative	Good
	6	PAGAWSPYLSSMSVWP	62	0.90	0.4729	Negative	Negative	Positive	Poor
	6	TNEVRADQKGGEGAST	269	0.90	1.9073	Negative	Negative	Positive	Good
	6	AARSEDRDEQDGETNK	208	0.90	1.4092	Negative	Positive	Positive	Good
TgRON10	1	GIHISERRTYGFKPNV	285	0.95	0.4261	Negative	Negative	Positive	Good
	2	PLAGPTPPEHHKDKGD	524	0.93	1.2937	Negative	Negative	Positive	Good
	2	CGSWQTVPSSPHFSSN	234	0.93	0.2662	Positive	Positive	Positive	Poor
	3	DRMWIPPVRHMHLDLR	391	0.92	0.9149	Negative	Positive	Positive	Good
	4	EKVCQWHLLRESCLVP	507	0.91	-0.6632	Negative	Positive	Positive	Good
	5	TESVETPVEKIGENSQ	722	0.90	0.7131	Negative	Positive	Positive	Good
	5	NFLYSQPVPPEFIITP	415	0.90	0.9922	Negative	Negative	Positive	Poor
TgRON13	1	KLSQMADPEPLARVSM	749	0.95	0.4023	Negative	Negative	Negative	Good
	1	EREEYSSLLFDAKLPE	1145	0.95	1.5061	Negative	Positive	Positive	Good
	2	FDTISQGGTPSAEQTD	377	0.94	0.3169	Negative	Negative	Positive	Good
	3	WGSQEETDERREDRQE	174	0.93	1.3270	Positive	Negative	Positive	Good
	4	**PTSSSAFRDMVRIADP**	407	0.90	0.6009	Negative	Negative	Negative	Good
	4	PAHLRSPSPARFGWPP	1205	0.90	0.6165	Negative	Positive	Negative	Poor

[1] VaxiJen score: Threshold for parasites: 0.5
[2] AlgPred: A hybrid approach (SVMc + IgE epitope + ARPs BLAST + MAST) was applied.

EXCLI Journal 2025;24:749-773 – ISSN 1611-2156
Received: February 24, 2025, accepted: June 25, 2025, published: July 10, 2025

Table S7: Predicted cytotoxic T lymphocyte (CTL) epitopes for six TgRON proteins using the NetCTL 1.2 server.

Protein	CTL epitopes								
	A2 supertype	Score	Immunogenicity	A3 supertype	Score	Immunogenicity	B7 supertype	Score	Immunogenicity
**TgRON2**	RAFLVLILL	0.7934	0.16244	SLFFSSFPR	1.5389	-0.06871	MTKRAGLPL	0.8426	0.03624
	FLVLILLSA	1.2442	0.01538	GLRPQPSPR	0.8490	-0.2637	LPLGRAFLV	0.8563	0.21005
	ILLSAADSL	1.0712	-0.17308	RTFRPTGYQ	0.8197	0.14226	FSSFPRSAL	0.9058	-0.01474
	SALQLFSSV	0.8670	-0.25704	LLQLKRMLR	1.1684	-0.36472	FPRSALQLF	1.4648	-0.22625
**TgRON4**	TLTGSGLLV	0.9103	-0.09834	RMYPNMNER	0.8570	-0.13092	SPPTPAPRM	0.7945	0.08237
	GLLVLLTLA	0.8515	0.04298	RSELHIDLK	0.8570	0.19036	APRMYPNMN	0.8873	-0.282
	LTLACGTTV	0.9198	0.07061	HIDLKSPQK	1.2305	-0.44673	YPNMNERPL	1.6762	-0.05365
	NMNERPLSA	0.8555	0.03259	VVTPIPASK	1.2035	0.05696	RPLSAESRL	1.3729	-0.1471
**TgRON4L1**	QVIPSGFEL	0.7600	0.06014	NLLTPAFRR	0.9882	0.19053	TPAFRRIVA	1.4972	0.36606
	ELIEEVPIV	0.8672	0.34871	TVTMPPRVK	0.9089	-0.11754	AVACAAAAL	1.3031	0.08926
	FQFAFRDGV	0.9439	0.27861	RVKAPPVPK	1.3509	-0.02351	PPRVKAPPV	1.0146	-0.13067
	TMRKLVAYI	0.8334	-0.14128	ATMRKLVAY	0.9925	-0.16766	APPVPKSYV	1.0403	-0.31764
**TgRON5**	LLMSLPKMI	0.8346	-0.52931	RPLLMSLPK	0.7801	-0.35733	MAEFTWRPL	1.0070	0.43381
	KMIAFFHIL	1.4094	0.41183	IAFFHILLF	0.7750	0.29674	RPLLMSLPK	0.8894	-0.35733
	MIAFFHILL	1.0817	0.38079	RQHGSGPPR	0.8751	-0.10044	RPAPRRAAA	1.7596	0.15654
	LLFSGALAA	1.0329	-0.04514	CLSRRKPTY	0.8778	-0.1409	APRRAAAVA	1.3745	0.20095
**TgRON8**	MVATTLHSL	1.0359	0.00976	TTLHSLPSR	0.9637	-0.24861	MVATTLHSL	1.1730	0.00976
	YLYTLLMSF	1.0350	-0.22824	TLHSLPSRY	1.2245	-0.28659	LPSRYLYTL	1.2977	0.0039
	TLLMSFLFV	1.1846	-0.17216	HSLPSRYLY	1.0727	-0.13674	MVSGFRASL	1.3830	0.07948
	LLMSFLFVC	0.8340	0.00301	RVTTQQMER	1.0725	-0.25988	YIRTAVHLL	0.9611	0.15364
**TgRON9**	MLSVFPESA	0.8351	0.07924	SVFPESARK	1.3840	0.03187	RPRFPPFFC	1.2227	0.28056
	SLGVSGATV	0.7547	-0.02096	ASLSPSLFR	1.3263	-0.27756	PPFFCPPSL	0.7609	-0.01316
	GVSGATVLL	0.7590	0.0834	LIASKSGGK	1.3056	-0.4711	GVSGATVLL	0.7839	0.0834
	VLLPFVGVL	1.0151	0.19082	KTGTDGHEK	0.7927	0.18936	LPFVGVLLF	0.9018	0.13556
**TgRON10**	RLRGASPLV	0.8620	-0.08257	LTLLVAAPK	1.2519	0.08881	SPLVSIFVL	1.3501	0.12504
	LVSIFVLTL	0.7542	0.2464	AIHHLGNNR	0.8684	0.05491	SPQWATATT	1.3140	0.31563

EXCLI Journal 2025;24:749-773 – ISSN 1611-2156
Received: February 24, 2025, accepted: June 25, 2025, published: July 10, 2025

	SIFVLTLLV	1.2130	0.08944	RSARADLVK	1.0298	0.13852	RPESSSWGF	1.4193	-0.24406
	TLLVAAPKL	0.9509	-0.02249	SSPHFSSNK	1.0836	-0.15618	SPSAPRFAA	1.4280	0.14525
**TgRON13**	FALRFLCPV	1.1769	0.10006	MLPRMQSER	0.8210	-0.31268	VARQSLGAT	0.9934	-0.21984
	FLCPVLCSL	1.3207	-0.14106	ATRSRCLLR	0.9041	-0.16586	GATRSRCLL	1.0200	-0.09968
	LLLATLFFV	1.4090	0.23033	RCLLRFATK	0.9846	0.20154	RPPPSSAAG	1.0222	-0.27571
	AAPPDSFFV	0.8471	0.01831	CLLRFATKK	1.5694	0.10607	CPLFPRSSF	1.5890	-0.08416

EXCLI Journal 2025;24:749-773 – ISSN 1611-2156
Received: February 24, 2025, accepted: June 25, 2025, published: July 10, 2025

Table S8: Helper T lymphocyte (HTL) epitope prediction for eight TgRON proteins, assessed for antigenicity and their potential to induce IFN-γ and IL-4 responses against the HLA reference set.

Protein	Allele	HTL epitope	Percentile rank	Antigenicity	IFN-γ inducing		IL-4 inducing	
					Result	Score	Result	SVM score
TgRON2	HLA-DQA1*01:01/DQB1*05:01	VPSGIRVYATPPAPR	0.01	0.3193	POSITIVE	0.2689	IL4-inducer	1.27
	HLA-DPA1*02:01/DPB1*05:01	PRTFRPTGYQRIEVK	0.01	0.5928	POSITIVE	1	IL4-inducer	0.48
	HLA-DQA1*01:02/DQB1*06:02	EGLIEAVQLRAAEKG	0.01	0.6162	POSITIVE	0.7448	IL4-inducer	0.31
TgRON4	HLA-DQA1*05:01/DQB1*03:01	PSSEAASAAAESSDE	0.02	1.2062	NEGATIVE	3	Non-IL4-inducer	-0.22
	HLA-DRB3*02:02	HQQWKKNKAEVSKLG	0.02	0.9823	NEGATIVE	1	IL4-inducer	0.32
	HLA-DQA1*03:01/DQB1*03:02	AQPAYETVYGDEEDR	0.02	1.2156	NEGATIVE	-0.2674	IL4-inducer	0.36
TgRON5	HLA-DRB4*01:01	SDNLTLQDIDINASS	0.01	0.2889	NEGATIVE	-0.1919	Non-IL4-inducer	0.03
	HLA-DRB1*08:02	RKKAFAAARKLKSLK	0.01	0.2718	POSITIVE	0.1959	IL4-inducer	0.51
	HLA-DRB1*04:01	IDKFKQDAAAAAEAA	0.01	0.4544	NEGATIVE	3	IL4-inducer	1.33
TgRON9	HLA-DQA1*03:01/DQB1*03:02	DDSVESAGPAADEQQ	0.01	1.5973	NEGATIVE	2	Non-IL4-inducer	0.09
	HLA-DRB3*02:02	AGNAIKVNVPNKNGK	0.01	0.7693	NEGATIVE	-0.3968	IL4-inducer	1.51

	HLA-DRB1*09:01	NTPLHYAAAFNADKS	0.03	0.3193	NEGATIVE	-0.4131	IL4-inducer	1.52
TgRON10	HLA-DQA1*01:02/DQB1*06:02	QEGATAAEAQPQEAK	0.01	1.1710	POSITIVE	1	Non-IL4-inducer	-0.54
	HLA-DQA1*01:02/DQB1*06:02	PSQEGATAAEAQPQE	0.01	1.2164	POSITIVE	0.0665	Non-IL4-inducer	-0.17
	HLA-DRB1*08:02	DQGFHAAIHHLGNNR	0.02	-0.2010	NEGATIVE	3	Non-IL4-inducer	0.13
TgRON13	HLA-DRB5*01:01	HEIFRKAIAFKKDIS	0.01	0.0118	POSITIVE	0.3167	IL4-inducer	1.30
	HLA-DRB4*01:01	GKDILKLVAVDPAAR	0.01	0.1796	NEGATIVE	7	Non-IL4-inducer	0.02
	HLA-DRB1*04:01	PAEYVRVADTEAVAP	0.01	0.4901	NEGATIVE	-0.0175	IL4-inducer	0.63
